# E-cigarettes as smoking cessation aids: a survey among practitioners in Italy

**DOI:** 10.1007/s00038-015-0772-x

**Published:** 2015-12-21

**Authors:** Lambros Lazuras, Milena Muzi, Caterina Grano, Fabio Lucidi

**Affiliations:** Department of Psychology, Sociology and Politics, Sheffield Hallam University, Sheffield, S10 2BQ UK; Department of Social and Developmental Psychology, ‘La Sapienza’ University of Rome, Rome, Italy

**Keywords:** Electronic cigarette, Smoking cessation, Safety, Beliefs

## Abstract

**Objectives:**

To describe experiences with and beliefs about e-cigarettes as safe and useful aids for smoking cessation among healthcare professionals providing smoking cessation services.

**Methods:**

Using a cross-sectional design, anonymous structured questionnaires were completed by 179 healthcare professionals in public smoking cessation clinics across 20 regions in Italy.

**Results:**

Service providers reported that considerably more smokers made inquiries about e-cigarettes in 2014 than in 2013. The most frequent inquiries concerned the ingredients, safety and effectiveness of e-cigarettes as smoking cessation aids. Clients used e-cigarettes to quit smoking, cut down the number of conventional cigarettes smoked, have a safe alternative to smoking, and protect their health while continuing to smoke. More than 60 % of service providers reported favourable beliefs about the safety and effectiveness of e-cigarettes, and believed that e-cigarettes are as effective as other smoking cessation aids, including pharmacotherapy.

**Conclusions:**

Despite limited empirical evidence, service providers in Italy viewed e-cigarettes, as safe and effective smoking cessation aids. More concerted efforts are needed to improve knowledge about e-cigarettes among service providers, to guide their clinical practice and decision-making with respect to e-cigarettes.

## Introduction

Electronic or vapour nicotine delivery systems such as e-cigarettes, have become increasingly popular across different populations and cultures over the last decade (Kuschner et al. 2011; Schivo et al. 2014). Recent surveys in different countries show that e-cigarette use is increasing among adults and adolescents (Dockrell et al. 2013; Douptcheva et al. 2013; Goniewicz and Zielinska-Danch 2012), and that e-cigarette sales may surpass sales of conventional cigarette in the next few years (Bhatnagar et al. 2014). Tobacco companies recently entered the e-cigarette market, promoting them as safe alternatives to conventional cigarettes, as effective cessation aids, and as safe products that enable smoking in public without contravening smoke-free policies (Grana et al. 2014). Smokers tend to respond favourably to e-cigarette marketing and believe that e-cigarettes are significantly less harmful than conventional cigarettes or other tobacco products such as smokeless tobacco and snus (Pepper et al. 2015). However, there is limited evidence about the side effects of e-vapour (McAuley et al. 2012), and it is not yet known if e-cigarette use is a gateway to the use of conventional cigarette leading to increased smoking initiation in younger populations (Dutra and Glantz 2014; Flouris and Oikonomou 2010; Pauly et al. 2007). Stated differently, even if exposure to e-cigarette vapour is safe, freely smoking e-cigarettes in public may promote more favourable social norms towards smoking, and this may run counter to public health campaigns which have de-normalized smoking in the last decades.

To guide public health policy and practice, public health policy makers need evidence-based information about the utility of e-cigarettes, especially in regard to cessation among current smokers and prevention among younger populations. Several studies support the effectiveness of e-cigarettes as smoking cessation aids (see Schivo et al. 2014). A prospective study among Italian smokers not willing to quit smoking, showed that e-cigarette use helped them reduce use of conventional cigarettes and remain abstinent over 24 weeks, with minor irritations in the throat and mouth that gradually dissipated (Polosa et al. 2011). A clinical trial in New Zealand among 657 adult smokers showed that e-cigarettes with nicotine were not more effective in smoking cessation than nicotine patches or placebo (non-nicotine) e-cigarettes (Bullen et al. 2013). A recent longitudinal study showed that smokers who used e-cigarettes daily for at least 1 month were six times more likely to quit smoking compared to those who used e-cigarettes less regularly (i.e. not on a daily basis; Biener and Hargraves 2015).

Increasing public interest in e-cigarettes as well as the marketing and sales practices of e-cigarette manufacturers increase pressure on providers of smoking cessation services to distribute evidence-based information to those wanting to quit, and also necessitates rapid informed decision-making by public health policy-makers (Künzli 2014; McKee 2014). The UK-based Centre for Smoking Cessation and Training (McRobbie 2014) produced an e-cigarette briefing in May 2014, to initiate discussion about practice guidelines on e-cigarettes and how to respond to smokers’ inquiries about e-cigarettes. The briefing mentions that service providers should “be open to electronic cigarette use in people keen to try them; especially those who have tried, but not succeeded, in stopping smoking using licenced stop smoking medicines” (p. 3). The briefing also includes a summary of the existing literature about the effectiveness of e-cigarettes as smoking cessation aids.

To date few studies describe the views of health professionals who provide smoking cessation services (herein labelled “smoking cessation service providers”) on e-cigarettes and their effectiveness in smoking cessation, as well as the types of inquiries that service providers respond to in their daily practice. A recent study in the UK showed that service providers held more positive attitudes about e-cigarette use in 2013 compared to baseline measures in 2011. In addition, the study reported an increase in the number of clients asking about e-cigarette products (Hiscock et al. 2014). Such information is important for several reasons. First, assessing service providers’ attitudes about e-cigarettes could uncover training needs and therefore inform education programmes and training for this professional group. Second, by investigating the type of inquiries received by smokers who seek smoking cessation advice, information toolkits can be developed and mentoring support provided to service providers to better respond to such inquiries and avoid misleading the public. This latter issue is important because the advertised alleged benefits of e-cigarettes may lead to false expectations about their safety and effectiveness in treating tobacco dependence. The present study aims to describe experiences and beliefs about e-cigarettes among smoking cessation service providers in Italy. Specifically, we investigated the type of inquiries service providers received about the use and safety of e-cigarettes, their experiences with their clients’ use of e-cigarettes, as well as their own beliefs about the safety and efficacy of e-cigarettes.

## Methods

A cross-sectional design was used, and data collection was completed between January and March 2014. All active public smoking cessation centres and clinics in Italy (*N* = 224) that are registered with the national health authority (Osservatorio Fumo and Alcol e Droga 2013), were contacted by telephone to complete the questionnaire. These clinics all provide the same services to their clients, although the specific treatment used may differ, all clinics included a combination of pharmacotherapy and behavioural counselling (Di Pucchio et al. 2009). Of the 224 centres, 179 in 20 districts across Italy (80 %) agreed to participate. The most frequently stated reason for non-participation was limited time and increased workload at the time of the study. One representative from each centre completed the questionnaire. All respondents were active smoking cessation practitioners; 52 % (*n* = 93) provided smoking cessation services on behalf of locally commissioned smoking cessation services, and 48 % (*n* = 85) provided smoking cessation services and also managed a locally commissioned smoking cessation service. ‘Managing’ refers to smoking cessation service providers who also organize clinical activities and tasks for subordinates and/or fellow clinicians. Only 19.6 % (*n* = 35) of respondents reported that providing smoking cessation services comprised all or most of their professional duties.

Completion of telephone-administered questionnaires was facilitated by trained researchers from La Sapienza University of Rome. All respondents were informed about the aims of the study and their participation rights (e.g. anonymity and confidentiality of responses, voluntary participation). Given that the smoking cessation centres might be identifiable by their location, no questions were asked about the sex or age of respondents to safeguard anonymity and confidentiality of responses.

### Measures

A structured questionnaire designed by smoking cessation experts from the National Centre for Smoking Cessation and Training in the UK (Hiscock et al. 2014), was translated and adapted to Italian. It included questions relevant to sources of information about e-cigarettes, frequency and type of inquiries about e-cigarettes, proportion of clientele who used e-cigarettes, reasons clients used e-cigarettes, clients’ experiences of e-cigarettes, and service providers’ beliefs about e-cigarettes. More specifically, service providers’ beliefs about e-cigarettes included five items reflecting general beliefs about e-cigarettes (e.g. ‘I think that e-cigarettes are a good thing’), and about their safety and efficacy for smoking cessation (e.g. ‘e-cigarettes are safe products to use’; ‘e-cigarettes are as effective as smoking cessation medication’). Responses were coded on a five-point Likert scale (1 = strongly agree, 5 = strongly disagree), and a mean score was computed. Lower scores reflected more positive attitudes about e-cigarettes. The internal consistency of the scale in this sample was acceptable (Cronbach’s *α* = .78).

## Results

### Frequency and content of inquiries about e-cigarettes

Approximately two-thirds (68.2 %) of respondents first heard about e-cigarettes through the media, 11.2 % had heard about them from their clients, and 7.8 % through other professional networks. Compared to 2013, 44 % reported that more clients made inquiries about e-cigarettes; and 40.8 % reported that more than one quarter of their clients made inquiries about e-cigarettes in the last 6 months.

Table 1 describes the type of inquiries made by the service providers’ clientele and service providers’ responses. The most common inquiries included whether or not e-cigarettes are effective in helping smokers quit (83.2 %), if they contain harmful additives (79.3 %), if e-cigarettes are safe to use (72.6 %), and whether they are effective in helping smokers cut down or avoid smoking (72.1 %). Only 28.5 % of smokers inquired about the potential harm of second-hand exposure to e-vapour.

**Table 1:** Type of inquiries received by smoking cessation service providers in 2014 in Italy, and reasons why clients used e-cigarettes (*n* = 179)

	%
Inquiries received about e-cigarettes	
Where to get them?	11.7
Are they effective in helping people stop smoking?	83.8
Do they contain harmful additives?	79.3
How safe are they for people who use them?	72.6
Are they effective in helping people cut down or avoid smoking?	72.1
How do they work?	34.6
How safe are they for people around those who use them?	28.5
Are they illegal?	19
Do stop smoking services provide e-cigarettes	15.6
Why e-cigarettes are not provided by stop smoking services	8.9
How much do they cost?	8.9
Reasons for which clients have used e-cigarettes	
To try to quit	70.9
To help them cut down the number of cigarettes they smoked	62
When they are unable to smoke	27.4
As an alternative to smoking	45.8
To protect their health	34.6
To see what they are like	25.1
To protect the health of those around them	8.9
To get rid of the smell of stale smoke	3.4
Clients who ever used e-cigarettes	
None	1.3
Less than a quarter	61.0
Quarter to a half	25.8
Half to three quarters	5.7
More than three quarters	6.3

### Prevalence of and reasons for using e-cigarettes among clients

One-quarter (25.8 %) of service providers reported a quarter to half of their clients had used e-cigarettes, and 5.1 % reported that a quarter to half used e-cigarettes regularly. Most of their clients who ever had used e-cigarettes did so because they wanted to try to quit (70.9 %), to help reduce the number of conventional cigarettes used (62 %), or as an alternative to smoking conventional cigarettes (45.8 %) (Table 1).

### Client experiences with using e-cigarettes

Overall, 70.4 % of service providers who responded in the survey agreed (agree/strongly agree) that their clients who used e-cigarettes thought that these had been useful in helping them to quit smoking. Accordingly, only 23.5 % agreed that their clients found e-cigarettes helpful in cutting down the number of conventional cigarettes, and 44.7 % agreed that their clients viewed e-cigarettes as useful alternatives to smoking conventional cigarettes.

### Service providers’ beliefs about e-cigarette use

Most service providers displayed favourable beliefs about e-cigarette use. A more detailed analysis of frequencies showed that 79.3 % agreed (agree/strongly agree) that e-cigarettes are equally effective to smoking cessation medication, 70.9 % agreed that e-cigarettes are effective aids to smoking cessation, 64.2 % agreed that e-cigarettes are good to use, and 62.6 % agreed that e-cigarettes are safe to use.

## Discussion

E-cigarettes are popular commercial products that are marketed as safe and effective aids to smoking cessation. The present study describes beliefs of Italian smoking cessation service providers about e-cigarettes, interest in e-cigarettes among their clients, and the type of inquiries they receive from their clientele about e-cigarettes. The number of inquiries about e-cigarettes in public smoking cessation clinics in Italy increased compared to previous years indicating increased public interest. Most inquiries about e-cigarettes related to their safety and effectiveness as smoking cessation aids. Interestingly, most Italian service providers believed that e-cigarettes are safe and as effective as conventional pharmacotherapy.

Although the settings differ, our data can be compared with a recent survey of service providers in the UK (Hiscock et al. 2014). More than 60 % of Italian service providers believed that e-cigarettes are safe products that can help smokers quit, and that they are as effective as pharmacotherapy. In contrast, the UK study (Hiscock et al. 2014) showed that, although attitudes about e-cigarettes have become more favourable, most service providers remained sceptical, maintaining that there is little empirical evidence to support effectiveness or safety, and that e-cigarette use increases visibility of public smoking and may therefore undermine existing tobacco control policies.

This difference aligns with the current debate among public health professionals, with some scholars recommending that e-cigarettes should be integrated in clinical practice, and others stating that e-cigarettes should be viewed with caution until more evidence becomes available (Grana et al. 2014; McKee 2014). Our findings underscore the need for more concerted action to improve knowledge among service providers in Italy with respect to the safety and efficacy of e-cigarettes. They must be better informed about recent evidence and incorporate this knowledge into their practice.

The present study showed that 25.8 % of service providers reported that a ‘quarter to half’ of their clients had used e-cigarettes (among clients who visited smoking cessation clinics, only 12 % reported e-cigarette use) and only 5.1 % reported that ‘quarter to half’ used e-cigarettes regularly. Hiscock et al. (2014) presented similar findings, and reported that compared to measures 1 year earlier, more UK service providers reported that a ‘quarter to half’ of their clients had used (40 %), or regularly used e-cigarettes (23.5 %). Based on our findings, the prevalence of e-cigarette use in Italy in 2014 seems to be lower than that reported in the UK by Hiscock et al. (2014). Our data in fact align with those reported in the annual report commissioned by the National Health Institute of Italy (Istituto Superiore di Sanita 2014), which showed a decrease of e-cigarette use between 2013 and 2014.

Our findings showed that 44 % of service providers reported that, compared to 2013, more clients asked about e-cigarettes, suggesting increased public interest in these products. In the UK, there was also an increase in client inquiries about e-cigarettes—90 % of providers reported that more clients made inquiries in 2013 compared to previous years (Hiscock et al. 2014).

Concordant with a recent study among Dutch smokers (Hummel et al. 2015), the most common types of inquiry received about e-cigarettes pertained to their efficacy and safety. Clients may be more concerned about the effects of e-cigarettes on their own health, than about the effects of e-cigarette vapour on others. This is not unexpected, but should be highlighted because it relates to questions about the public safety.

The public health policy implications of the present study are simple, but profound. First, policy-makers should consider if the public use of e-cigarettes undermines existing smoke-free policies that aim to prevent smoking among young people, protect non-smokers from the harmful exposure to second-hand smoke and possibly from exposure to e-vapour, and to de-normalize tobacco use in public places. These concerns are echoed among public health researchers in other countries. Commenting on the increase of e-cigarette use in South Korea, Zhu et al. (2014) argued that the unregulated use of e-cigarettes in public places may be a way for smokers to circumvent smoke-free policies. Based on our findings, we argue that smoking cessation service providers should keep in mind the potential effects of their advice on the wider public about exposure to e-vapour and the possibility that e-cigarette use may act as a gateway to smoking initiation among young people. Additionally, our study indicates that there is a need for comprehensive training of service providers about the safety and effectiveness of e-cigarettes as a smoking cessation treatment. Such training will improve smoking cessation service providers’ knowledge about e-cigarettes, and, accordingly, help them in making informed decisions about e-cigarettes in the course of treating their patients. Currently, there is a noted expansion of e-cigarette products and most of the available information about e-cigarettes comes from the promotional campaigns of the e-cigarettes industry (Zhu et al. 2014). If smoking cessation service providers serve the purpose of impartially informing the public about e-cigarette use, then comprehensive evidence-based training about e-cigarettes will provide a significant added value to their daily practice.
